# Gene Expression Profiling Identifies Molecular Pathways Associated with Collagen VI Deficiency and Provides Novel Therapeutic Targets

**DOI:** 10.1371/journal.pone.0077430

**Published:** 2013-10-11

**Authors:** Sonia Paco, Susana G. Kalko, Cristina Jou, María A. Rodríguez, Joan Corbera, Francesco Muntoni, Lucy Feng, Eloy Rivas, Ferran Torner, Francesca Gualandi, Anna M. Gomez-Foix, Anna Ferrer, Carlos Ortez, Andrés Nascimento, Jaume Colomer, Cecilia Jimenez-Mallebrera

**Affiliations:** 1 Neuromuscular Unit, Neurology Department, Fundación Sant Joan de Déu, Hospital Materno-Infantil Sant Joan de Déu, Barcelona, Spain; 2 Bioinformatics Core Facility, Institut d'Investigacions Biomediques August Pí i Sunyer (IDIBAPS), Barcelona, Spain; 3 Pathology Department, Hospital Materno-Infantil Sant Joan de Déu, Barcelona, Spain; 4 Dubowitz Neuromuscular Centre, MRC Centre for Neuromuscular Diseases, University College London Institute of Child Health, London, United Kingdom; 5 Pathology Department, Hospital Virgen del Rocío, Sevilla, Spain; 6 Orthopaedic Surgery & Traumatology Department, Hospital Materno-Infantil Sant Joan de Déu, Barcelona, Spain; 7 Dipartimento di Riproduzione e Accrescimento, U.O. di genetica Medica, Azienda Ospedaliero-Universitaria S'Anna, Ferrara, Italy; 8 Department of Biochemistry and Molecular Biology & Center for Biomedical Research on Diabetes and Metabolic Disorders (CIBERDEM), University of Barcelona, Barcelona, Spain; 9 Microarray Unit, Centro de Regulación Genómica, Barcelona, Spain; 10 Center for Biomedical Research on Rare Diseases (CIBERER, ISCIII), Madrid, Spain; University of Valencia, Spain

## Abstract

Ullrich congenital muscular dystrophy (UCMD), caused by collagen VI deficiency, is a common congenital muscular dystrophy. At present, the role of collagen VI in muscle and the mechanism of disease are not fully understood. To address this we have applied microarrays to analyse the transcriptome of UCMD muscle and compare it to healthy muscle and other muscular dystrophies. We identified 389 genes which are differentially regulated in UCMD relative to controls. In addition, there were 718 genes differentially expressed between UCMD and dystrophin deficient muscle. In contrast, only 29 genes were altered relative to other congenital muscular dystrophies. Changes in gene expression were confirmed by real-time PCR. The set of regulated genes was analysed by Gene Ontology, KEGG pathways and Ingenuity Pathway analysis to reveal the molecular functions and gene networks associated with collagen VI defects. The most significantly regulated pathways were those involved in muscle regeneration, extracellular matrix remodelling and inflammation. We characterised the immune response in UCMD biopsies as being mainly mediated via M2 macrophages and the complement pathway indicating that anti-inflammatory treatment may be beneficial to UCMD as for other dystrophies. We studied the immunolocalisation of ECM components and found that biglycan, a collagen VI interacting proteoglycan, was reduced in the basal lamina of UCMD patients. We propose that biglycan reduction is secondary to collagen VI loss and that it may be contributing towards UCMD pathophysiology. Consequently, strategies aimed at over-expressing biglycan and restore the link between the muscle cell surface and the extracellular matrix should be considered.

## Introduction

Ullrich Congenital Muscular Dystrophy (UCMD, OMIM #254090), caused by collagen VI deficiency, is one of the most common inherited myopathies [1]. It is characterized by hypotonia, delayed motor milestones, proximal muscle weakness, distal joint hyperlaxity and proximal joint contractures within the first year of life. Patients at the severe end of the of UCMD spectrum never achieve ambulation whereas most patients walk independently but loose ambulation around the early teenage years. Feeding difficulties in childhood are also a relatively common feature, as respiratory insufficiency which is almost invariable by the late teens. Furthermore, a characteristic skin phenotype has been described in collagen VI-related disorders [2,3][4]. 

UCMD is caused by recessive or dominant mutations in any of the three collagen 6 genes (*COL6A1*, *COL6A2* and *COL6A3*) which result in an absence or partial deficiency of collagen VI around the muscle fibre. Collagen VI is secreted by endomysial fibroblasts [5] and consists of three α chains, namely α 1(VI), α 2(VI) and α 3(VI). They form heterotrimeric monomers that align to form antiparallel dimers which align laterally to form tetramers. These tetramers are secreted from the fibroblast and associate in an end-to-end fashion to give rise to the final microfilament network [2]. Collagen VI microfibrils interact with various components of the muscle basal lamina and extracellular matrix and may also interact directly with the muscle cell via yet unknown surface receptors.

The function of collagen VI in skeletal muscle is still not fully understood. Its position in close proximity to the basal lamina, suggests that it acts by linking the basal lamina to the extracellular matrix [6]. Previous works in cell cultures has shown that collagen VI-deficient cells exhibit decreased adherence to their surroundings [7,8]. Collagen VI deficiency has also been associated with alterations of the mitochondrial membrane permeability, which lead to increased cell death by apoptosis, and with impaired autophagy supporting non-structural roles in muscle as in other cell types [9-11]. Recent work has linked defects in autophagic removal of mitochondria (mitophagy) to muscular dystrophy associated with collagen VI deficiency [12]. Defective mitophagy permits the accumulation of damaged mitochondria that leads to mitochondrial swelling, collapse of the mitochondrial membrane potential, and release of pro-apoptotic factors, which have been implicated not only in cell death but also in muscle fibre atrophy and injury [13,14].

At present, there are no available treatments for UCMD although several strategies are being developed. Currently, there is an open pilot trial with cyclosporine A, an immunosuppressive agent, that seems to correct mitochondrial dysfunction observed in muscles from UCMD patients ameliorating the symptoms [15,16]. Another study is based on the use of anti-apoptotic drugs in collagen VI and laminin-α2 deficiency [17]. However, there are concerns over the use of anti-apoptotic and pro-autophagy treatments because of their pleiotropic and potentially harmful effects. Therefore, identifying more upstream modifiers may provide therapeutic targets which are more specific and with less unwanted consequences. 

Previous studies have used microarray technology to identify and describe molecular pathways implicated in other muscular pathologies such as Duchenne Muscular Dystrophy (DMD) [18-20][21-23], Limb-Girdle Muscular Dystrophy 2A (LGMD2A) [24,25], Emery-Dreifuss muscular dystrophy (EDMD) [26], facioscapulohumeral muscular dystrophy (FSHD) [27], X-linked myotubular myopathy (XLMTM) [28] and inflammatory myopathies [29] amongst others. 

Previously, we have described that collagen VI deficiency leads to concomitant and significant muscle cell atrophy and regeneration [30] although the precise mechanism remained to be elucidated. We sought to gain insight into genes and pathways involved in this disease and increase our understanding of the pathogenesis of this condition by using for first time, gene expression arrays and comparing gene expression patterns between UCMD, control and other muscular dystrophies. Using this approach we have identified significant changes in the transcriptome of UCMD relative to normal and dystrophin deficient muscle. We describe significant muscle regeneration, ECM remodelling and an inflammatory component which is mediated by M2 macrophages and the complement system. Furthermore, we have identified biglycan as a potential molecular target for future therapeutic development and clinical research. 

## Material and Methods

### Ethics Statement

This work has been approved by the Ethical Committee of “Fundació Sant Joan de Déu”. Written informed consent for research was obtained from all patients and healthy controls (or their parents/guardians) according to the Hospital Sant Joan de Déu forms and regulations. 

### Study Population

Open muscle biopsies from left quadriceps were performed, oriented and frozen according to standard procedures [31]. Muscle tissue samples for microarray analysis were recruited from 16 individuals: 6 UCMD patients, 3 DMD patients, 3 CMD patients and 4 non pathological children. Lower limb muscle samples were obtained from children non-affected by a neuromuscular disease (control muscle group) who underwent orthopaedic surgery at the Hospital Sant Joan de Déu. 

Additional samples of UCMD patients were supplied by MRC CNMD Bio Bank (London, UK), corresponding to patients P6 and P7, and by Hospital Virgen del Rocío (Sevilla, Spain), patient P8. P9 was not included in the microarray analysis but was studied by immunfluorescence. Detailed information on all patients and controls is given in Table S1. 

### RNA isolation and First-strand cDNA Synthesis

Muscle biopsies were homogenized with TissueRuptor (Qiagen, Hilden, Germany). Total RNA was extracted from biopsies (30 mg) with RNeasy Fibrous Tissue mini kit following the manufacturer’s instructions. Quantity and quality of RNA obtained was determined with Nanodrop 8000 Spectrophotometer (Thermo Scientific, Schwerte, Germany) and its integrity with Agilent 2100 Bioanalyzer (Agilent technologies, Waldbronn, Germany). In all samples RNA integrity numbers (RIN) were >7 and 260/280 ratios near 2.0.

0.15 mg of RNA was retro-transcribed with SuperScritp III First-Strand Synthesis Super-Mix for qRT-PCR (Invitrogen™, Carlsbad, CA, USA) to obtain cDNA and then was amplified with Taqman PreAmp Master Mix (Applied Biosystems, Foster City, CA, USA) following manufacturer’s instructions. 

### Microarray Analysis

RNA from muscle biopsies was amplified by MEGAScript T7 (Invitrogen™, Carlsbad, CA, USA) and retrotranscribed to cDNA and labelled with the IVT kit. A total of 15 μg of labelled cRNA was fragmented and hybridised to oligonucleotide Agilent GE 1-color Human 8x60 K arrays (Agilent Technologies, Inc., Santa Clara, CA, USA). The current format of these arrays interrogates 27,958 annotated genes in the human genome and 7,419 lincRNAs (long intergenic noncoding RNAs). Washes and scanning of the arrays were performed according to manufacturer instructions. Expression values of the 16 cy3-labeled samples were obtained using the Bioconductor *limma* package [32], with background correction ("normexp") and "quantiles" normalization between arrays.

Statistical differential gene expression analysis between groups was performed by the non-parametric approach *Rank Prod* [33], which detects genes that are consistently highly ranked in a number of replicate experiments, a method that has shown robustness to outliers, being suitable for studies with few biological samples. Those oligonucleotides that present changes between groups with FDR (False Discovery Rate) lower than 0.05 were considered significant. The web tool DAVID [34], was used for the calculation of the functional over-representation statistics of the different lists of significant genes obtained with *Rank Prod* analysis. Gene Ontology Biological Process (The Gene Ontology Consortium, www.geneontology.org) and KEGG pathways (Kyoto Encyclopedia of Genes and Genomes, www.genome.jp/kegg) databases were considered. Also, interaction networks have been constructed using the Ingenuity Pathways Analysis tool (IPA) (http://www.ingenuity.com), based on extensive records maintained in the Ingenuity Pathways Knowledge Base (IPKB) database. 

### Real-Time Quantitative RT-PCR

High-throughput real-time qPCR was performed according to the manufacturer's protocol on the BioMark 48.48 Dynamic Array (Fluidigm^®^, South San Francisco, CA, USA) with Taqman Gene Expression Assays. List of Taqman Gene Expression Assays used is provided in Table S2. qRT-PCR was run in triplicates in all samples and TATA box binding protein (*TBP*) and hypoxanthine phosphoribosyltransferase 1 (*HPRT1*) were used as endogenous control genes to normalize transcription levels amongst patients. Results were analysed with qBase^plus^ software (Biogazelle, Zwijnaarde, Belgium). Fold-changes of the genes of interest were calculated as mean values of 2^-∆∆CT^ or 1/2^-∆∆CT^ relative to healthy controls. A fold-change above or below 1.5 was considered significant. 

### Antibodies and Immunostaining

Cryosections (7 μm thick) were labelled without prior fixation using primary antibodies diluted in PBS 0.1 % Tween-20. Detailed information on primary antibodies used is provided in Table S3. Immunofluorescence was visualised with species-specific secondary antibodies directly linked to Alexa-fluorophores (Molecular Probes, Eugene, OR, USA). Nuclei were visualised using DAPI (Molecular Probes, Eugene, OR, USA) diluted in PBS 0.1% Tween-20. Immunohistochemistry was performed using Novo-Link peroxidase detection kit according to manufacturer’s instructions (Novo-Link peroxidase detection kit, Leica Microsystems, Wetzlar, Germany). Working dilutions were optimised for each antibody and non-specific labelling was assessed using sections incubated without primary antibodies.

Immunofluorescence was visualised under a conventional epifluorescence microscope (Leica DM5000B) or under a confocal microscope (TCS-SP2 Leica confocal microscope). Images were acquired with Leica Imaging Suite (LAS) and were quantified with ImageJ software. 

### Quantitation of ﬁbrosis

The extent of connective tissue was measured on collagen VI immunostained sections as previously described [35] using the NIH Image software version 1.44 (http://rsb.info.nih.gov/nih-image/). At least six ﬁelds from each patient were analysed. The area positive for collagen VI was calculated as a percentage of the entire image. The mean and standard deviation (SD) was then obtained for control and each disease group from the total of all analysed ﬁelds.

### Statistical analysis

Average values are expressed as mean ± standard error (MEAN ± SE). Significance was tested by Student unpaired *t*-test and *P*-value <0.05 was considered as significant (NS, non-significant, *P < 0.05, **P < 0.01 and ***P < 0.001). 

## Results & Discussion

### Overview

We used Agilent GE 1-color Human 8X60K microarrays to identify differences in gene expression between UCMD muscles, control muscles and other muscular dystrophies. 

This approach has been used before to describe the molecular mechanisms involved in other muscle diseases such as DMD [18-21][36], LGMD2A [24,25], FSHD [27], Emery-Dreifuss muscular dystrophy (EDMD) [26], X-linked myotubular myopathy [28] and inflammatory myopathies [29]. 

UCMD and other myopathy patients and controls were adequately differentiated by non-supervised hierarchical clustering analysis, showing considerable heterogeneity, whereas no mutation, age or genre effects were observed in the whole dataset. Then, we proceeded to perform the supervised statistical analysis with a method –RankProd- which has largely shown important effectiveness detecting genes that are consistently highly ranked in a number of replicate experiments, even when a small number of heterogeneous replicates are available. We identified 389 individual genes whose expression levels were significantly changed in UCMD muscles with respect to control muscles using the selection criteria of False Discovery Rate (FDR) < 0.05. A full gene list is provided in Table S4. Of these, a majority 319 genes were up-regulated and only 70 genes were down-regulated (Table 1). The top 10 significant differentially expressed genes are listed in Table 2. The list of up-regulated genes included some involved in regeneration and differentiation (*MYH8*, myosin heavy chain perinatal; *S100B*, S100 calcium binding protein beta), genes involved in the formation of lipid droplets and ketogenesis (*CIDEA* and *CIDEC*, cell death-inducing DFFA-like effector A and C, *PLIN1*, perilipin), genes encoding adipokines (*RBP4*, retinol binding protein 4; *ADIPOQ*, adiponectin) and various galectins (*LGALS7*, galectin-7; *LGALS12*, galectin-12). In the list of down-regulated genes were included those involved in protein synthesis (*IGFN1*, immunoglobulin-like and fibronectin type III domain containing 1), cell cycle and cell survival (*GADD45G*, growth arrest and DNA-damage-inducible, gamma; *AKR1B10*, aldo-ketoreductase family 1 member B10), transducers of intracellular signals (*ARRDC2*, arrestin domain containing 2), secreted proteases (*ADAMTS8*, ADAM metallopeptidase with thrombospondin type 1 motif 8), TNF-receptors (*RELT*, tumor necrosis factor receptor), sarcomeric components of the Z-disk (*ACTN3*, actinin alpha 3) and others genes that codify proteins of unknown function (*KLHL34*, kelch-like 34; *ZMYND17*, zinc finger MYND domain containing 17). Data has been deposited at the National Centre for Biotechnology Information Gene Expression Omnibus (GEO) database as GEO Series accession number GSE43698. 

**Table 1:** Number of regulated genes in UCMD vs control muscle, DMD and CMD.

	UCMD vs Control		UCMD vs DMD		UCMD vs CMD	
	Down	Up	Down	Up	Down	Up
FDR < 0.05	70 (118)	319 (658)	421 (552)	297 (643)	29 (38)	-
FDR < 0.01	8 (13)	116 (241)	52 (62)	57 (111)	13 (15)	-
FDR < 0.05 & IPA SKM	53	257	211	258	21	-

Numbers in brackets represent numbers of oligonucleotides regulated in the microarray. FDR, False Discovery Rate. IPA SKM, filtered for expression in skeletal muscle by Ingenuity Pathway Database.

**Table 2:** Top ten changes in UCMD vs Control.

**Gene symbol**	**Gene description**	**FC**	**FDR**
IGFN1	Immunoglobulin-like and fibronectin type III domain containing 1	-8.8	0
GADD45G	Growth arrest and DNA-damage-inducible, gamma	-4.6	0.0011
KLHL34	kelch-like 34	-4.1	0.0011
AKR1B10	Aldo-keto reductase family 1, member B10 (aldose reductase)	-3.7	0.002
ARRDC2	Arrestin domain containing 2	-4.1	0.0027
ZMYND17	MSS51 mitochondrial translational activator homolog	-3.3	0.0058
ADAMTS8	ADAM metallopeptidase with thrombospondin type 1 motif, 8	-4.1	0.0071
RELT	Tumor necrosis factor receptor	-3.1	0.01
ACTN3	Actinin alpha 3	-3.0	0.01
HPDL	4-hydroxyphenylpyruvate dioxygenase-like	-3.0	0.01
MYH8	Myosin, heavy chain 8, skeletal muscle, perinatal	57.7	0
RBP4	Retinol binding protein 4	19.6	0
CIDEC	Cell death-inducing DFFA-like effector c	11.6	0
LGALS7	Galectin 7	10.9	0
ADIPOQ	Adiponectin	9.6	0
PLIN1	Perilipin 1	9.4	0
S100B	S100 calcim binding protein B	8.0	0
HMGCS2	3-hydroxy-3-m3thylglutaryl-CoA synthase 2	7.5	0
HLA-DQA1	Major histocompatibility complex, class II, DQ alpha 1	5.3	3.33E-04
LEP	Leptin	5.1	3.33E-04

FC, Fold-change; FDR, False Discovery Rate.

In order to understand the biological significance of the observed changes we analyzed the differentially expressed UCMD muscle genes using DAVID Bioinformatics Resources (v6.7) web tool [34,37] that provides information of gene ontology (GO) classification and KEGG pathways, identifying significantly enriched themes. 

**Figure 1:**
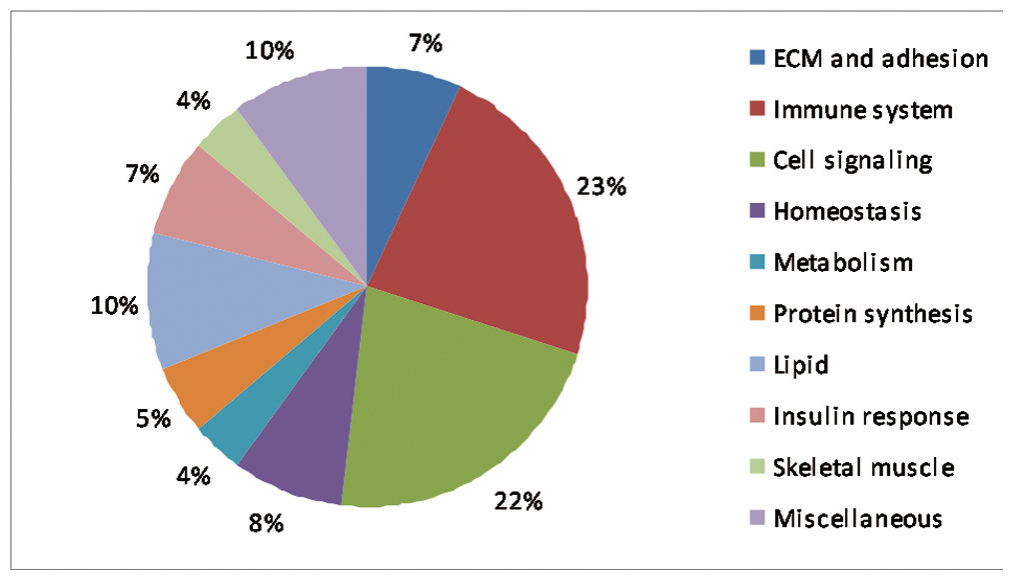
GO BP categories in UCMD vs control muscle. Pie chart representing GO BP categories up-regulated in UCMD vs control muscle.

Only GO BP (Gene Ontology Biological Process) categories with % FDR<20 were considered relevant. We found 146 significant GO BP categories of up-regulated genes (listed in Table S5) which were grouped in more general functional categories as shown in Figure 1. Among the most enriched biological processes of up-regulated genes were those related to the immune response, extracellular matrix and adhesion, regeneration, cell signalling and lipid and glucose metabolism; whereas only 6 significant GO categories of down-regulated genes were described including genes involved in catabolic processes (Table 3).

**Table 3:** GO categories of significant down-regulated genes.

**GO term**	**p-value**	**Genes**	**FDR**
Posttranscriptional regulation of gene expression	0.00338724	NANOS1, MKNK2, PEX6, GDNF, SAMD4A	4.800500143
Modification-dependent protein catabolic process	0.00638154	NEDD4, USP38, FBXO32, ASB2, TRIM63, CISH, USP31	8.864571736
Modification-dependent macromolecule catabolic process	0.00638154	NEDD4, USP38, FBXO32, ASB2, TRIM63, CISH, USP31	8.864571736
Proteolysis involved in cellular protein catabolic process	0.00787657	NEDD4, USP38, FBXO32, ASB2, TRIM63, CISH, USP31	10.83270743
Cellular protein catabolic process	0.00806415	NEDD4, USP38, FBXO32, ASB2, TRIM63, CISH, USP31	11.07683549
Protein catabolic process	0.00932847	NEDD4, USP38, FBXO32, ASB2, TRIM63, CISH, USP31	12.70613535

FDR, False Discovery Rate.

Concordant results were obtained when using KEGG database, although only up-regulated genes showed a significant association with KEGG pathways. A full pathway list is given in Table S6. 

Increased susceptibility to apoptosis, impaired activation of autophagy and accumulation of dysfunctional mitochondria and sarcoplasmic reticulum have been implicated in the pathogenesis of UCMD in both mice and human [9,12]. Therefore we looked for enrichment of these and related molecular functions amongst the set of regulated genes in UCMD versus control muscle using GO and KEGG. We did not find any significantly over or under represented GO_BP or canonical pathway associated with apoptosis, mitochondria or autophagy suggesting that these processes are not regulated at the gene transcription level. In contrast we found 2 GO_BP categories related to calcium homeostasis. 

### IPA analysis

**Figure 2:**
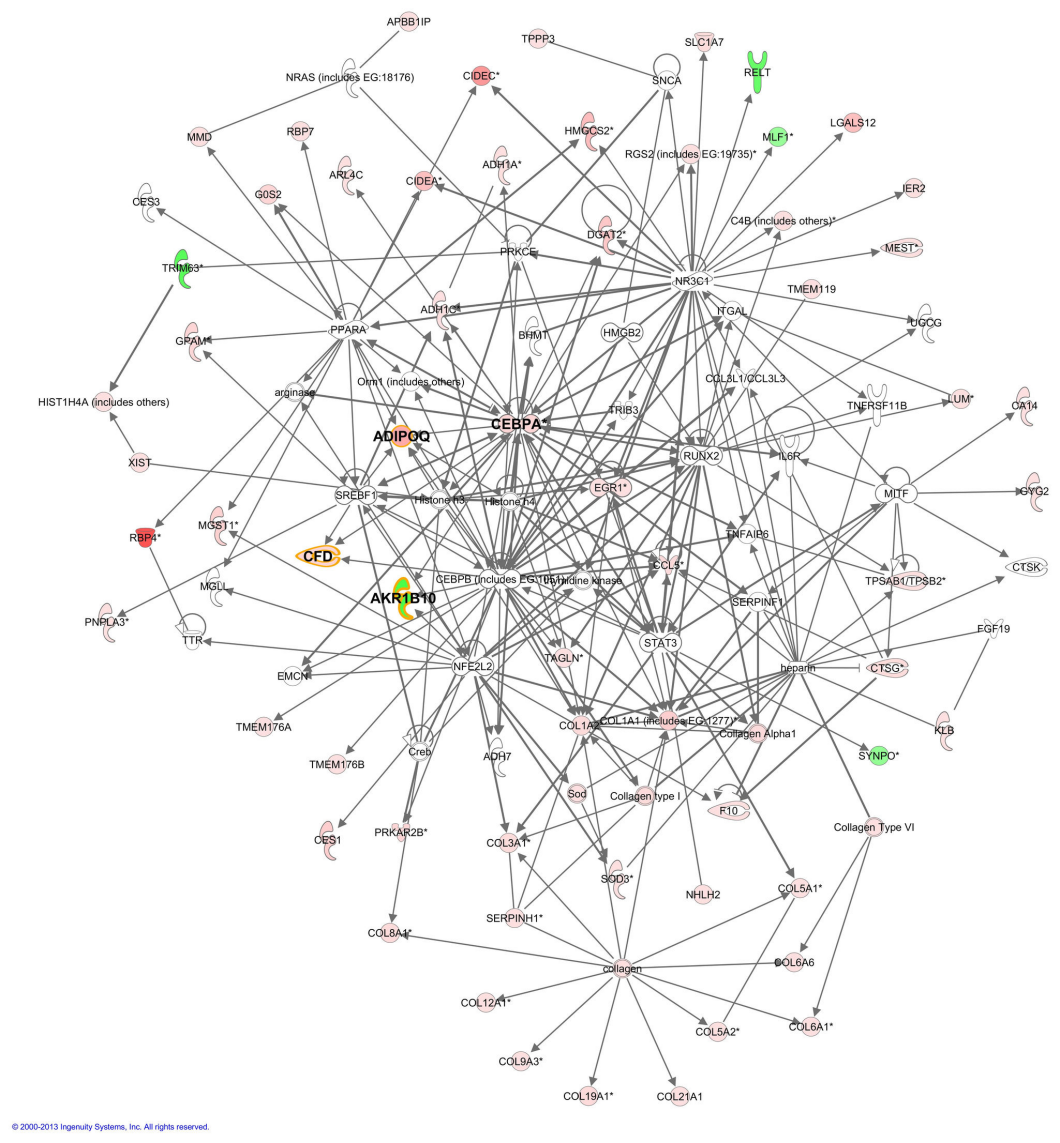
Merging of three well scored IPA networks of regulated genes in UCMD patients. Gene networks involved in Connective Tissue Disorders, Dermatological Diseases and Conditions, Gastrointestinal Disease (network 2, Table S4); Lipid Metabolism, Molecular Transport, Small Molecule Biochemistry (network 6); and Inflammatory Response, Cellular Movement, Haematological System Development and Function (network 8). The intensity of the node color indicates de degree of up-(red) or down- (green) regulation in UCMD compared to control. Genes in uncoloured notes were not identified as differentially expressed in our experiment and were integrated into the computationally generated networks on the basis of the evidence stored in the IPA knowledge database indicating a relevance to this network. Nodes are displayed using various shapes that represent the functional class of the gene product.

In order to corroborate which are the most significant biological functions revealed before by enrichment analysis and to identify novel relationships among genes associated with UCMD, all the significantly regulated genes were subjected to IPA^®^ analysis. This software revealed the signalling and metabolic pathways and biological process that are most significantly perturbed in UCMD (Table 4). Besides, 10 well ranked gene networks were derived by this tool (Listed in Table S7). The best ranked ones were those related to connective tissue disorder, lipid metabolism and inflammatory disease. IPA also identified 34 activated and 8 inhibited upstream transcriptional regulators in the list of significant genes suggesting that collagen VI plays a role in the regulation of gene transcription. Three of these genes were also significantly over-expressed in the UCMD group, supporting the importance of their function: *PPARG*, *CEBPA* and *EGR1*. Previous works showed some evidences that soluble collagen VI plays a role in proliferation by up-regulating cyclins A, B, and D1 [38] and survival through down-regulation of the proapoptotic Bax protein [39] of fibroblasts and in the regulation and differentiation of adipocytes [40]. Besides, other components of the ECM such as tenascin X have been described to regulate gene expression [41,42]. We represented the merging of three interesting mechanistic networks (2, 6 and 8: connective tissue disorder, lipid metabolism and inflammatory response, respectively) to describe plausible connections among upstream regulators and the observed gene expression changes and to point out the tight relationship between them (Figure 2). The outstanding hub genes connecting these 3 networks were adiponectin (*ADIPOQ*, +9.60), adipsin (*CFD*, +4.60) and an aldo-keto-reductase (*AKR1B10*, -3.7). 

**Table 4:** IPA functional analysis performed with UCMD vs control significant genes.

**Category**	**Top functions**	**p-value**	**# genes**
Diseases and disorders			
1	Cancer	3.48E-34 - 1.36E-04	200
2	Reproductive System Disease	6.38E-24 - 1.36E-04	127
3	Connective Tissue Disorders	3.68E-20 - 1.93E-05	110
4	Skeletal and Muscle Disorders	3.68E-20 - 1.15E-04	130
5	Inflammatory Disease	1.04E-19 - 6.83-05	110
Molecular and Cellular Functions			
1	Cellular Movement	1.01E-16 - 1.44E-04	101
2	Cell-to-Cell Signaling and Interaction	4.46E-14 - 1.26E-04	86
3	Lipid Metabolism	7.97E-13 - 8.07E-05	72
4	Small Molecule Biochemistry	7.97E-13 - 1.44E-04	98
5	Cell Death and Survival	1.66E-12 - 1.15E-04	125
Physiological System Development and Function			
1	Immune Cell Trafficking	1.01E-16 - 1.44E-04	77
2	Hematological System Development and Function	1.62E-15 - 1.44E-04	109
3	Connective Tissue Development and Function	3.74E-12 - 1.44E-04	87
4	Tissue Morphology	3.74E-12 - 1.15E-04	114
5	Organismal Development	1.92E-09 - 9.96E-05	107

We focused on a selection of representative aspects of UCMD pathophysiology (muscle regeneration, extracellular matrix remodelling, immune response and protein synthesis) for further studies. 

### Muscle regeneration & maturation

In muscle from UCMD patients we observed the post-natal expression of developmental isoforms of sarcomeric proteins such as embryonic myosin (*MYH3*, +4.12), perinatal myosin (*MYH8*, +57.65) and cardiac troponin T2 (*TNNT2*, +6.87). This would be in line with the persistent expression of perinatal myosin in a significant percentage of fibres in UCMD muscle (up to 30%) as we and others have demonstrated by immunohistochemistry [43][30].We also observed an over-expression of genes encoding components of the cytoskeleton such as different isoforms of myosin light chains (*MYL5*, +3.71; *MYL9*, +2.33) and other cytoskeletal elements (*ACTG*, +3.11; *TUBB6*, +2.33) supporting chronic remodelling of the cytoskeleton [18]. However, *PAX7* (which is expressed by satellite cells) and early myogenic markers (*MYOD, MYOG, CD56* and *CDH15*) were not amongst the regulated genes in UCMD versus normal muscle. 

We sought to identify the regenerative mechanisms active in UCMD skeletal muscle based on the gene expression data. Insulin pathway is one of the most important mechanisms regulating regeneration and size of the muscle fibre [44]. Insulin –like growth factor I (*IGF-I*), whose expression is known to be up-regulated in regenerating muscle in vivo, positively regulates the proliferation and differentiation of satellite cells/myoblasts in vitro via the Akt/PKB pathway [45] and calcineurin [46]. In line with this, in the present study we observed that *IGF-I* and its isoform *IGF-II*, were up-regulated in UCMD muscle (+2.50 and +2.60, respectively).

Additionally, follistatin (*FST*) was up-regulated in UCMD muscle (+4.10). Over-expression of this gene may promote muscle regeneration via blockage of myostatin signalling [47]. 

Furthermore, one of the most up-regulated genes was *S100B* (+8.00), a calcium-binding protein of the EF-hand type. It has been previously described that this protein stimulates myoblasts proliferation but inhibits myogenic differentiation and myotube formation [48-51], lending support to the suggestion that incomplete maturation may play a part in the pathogenesis of UCMD [43] [30]. 

### Extracellular Matrix remodelling

UCMD patients showed increased expression of a large number of genes encoding Extracellular Matrix (ECM) components. In order to find out if these changes were associated with an increase in fibrosis we quantified endomysial and perimysial fibrotic area in control, DMD and UCMD muscle by calculating percentage of the area occupied by Collagen VI with ImageJ software as reported previously [35]. None of the patients included in this study showed complete absence of collagen VI. They all showed a partial reduction at the basal lamina but normal expression in the endomysium which allowed us to quantify fibrotic area. We determined that in UCMD there was an increase of connective tissue which was comparable to the extent of fibrosis found in early phases of DMD (9.82 ± 2.07; 25.83 ± 1.28 and 22.61 ± 1.85 % of fibrosis per area; in control, DMD and UCMD, respectively) (p = 0.087) (Figure S1). 

Corresponding with the level of fibrosis in UCMD, the principal ECM forming collagens (types I and III) were up-regulated (+5.48 and +3.85, respectively). Type V collagen, another fibril-collagen that associates with types I and III, was also up-regulated (+3.26) as well as collagens with interrupted triple helices (FACITs), types XII (+2.70), XIV (+3.34), XIX (+4.13) and XXI (+3.16) whose function is to link fibrillar collagen to other ECM components and some of them, XII and XIV, which are expressed during skeletal muscle development. Short chain collagen that forms hexagonal networks, type VIII, was also up-regulated (+4.14). Recently, three novel collagen VI alpha chains (α4, α5 and α6) have been described although only *COL6A6* is expressed in human skeletal muscle [52]. Most of our UCMD patients presented *COL6A1* mutations and, as previously have been reported in skin, *COL6A6* was altered (+2.37) [53]. Changes in *COL6A6* have also been reported in Duchenne muscular dystrophy suggesting that it is implicated in muscle fibrosis [54]. 

Biglycan (*BGN*) and lumican (*LUM*) are members of the small leucine-rich proteoglycan (SLRP) family of ECM proteoglycans. Both genes were over-expressed in UCMD skeletal muscle (+2.26, +3.17). Lumican has been previously identified to interact with fibrillar collagens and can interfere with collagen fibrillogenesis. It has a stimulatory effect on the epithelial-mesenchymal transition state to fibrosis induced by transforming growth factor β (TGF-β). Biglycan, in addition to binding to fibrillar collagens I and III, interacts directly with collagen VI [55,56] [57] and is known to form a connection between the extracellular collagenous matrix and the dystrophin-associated protein complex (DAPC) via its binding to α-dystroglycan [58], α-sarcoglycan and γ-sarcoglycan [59]. It is also considered a signalling molecule because it interacts with growth factors and their receptors and recently it has been established as a part of the innate immune system [60].

**Figure 3:**
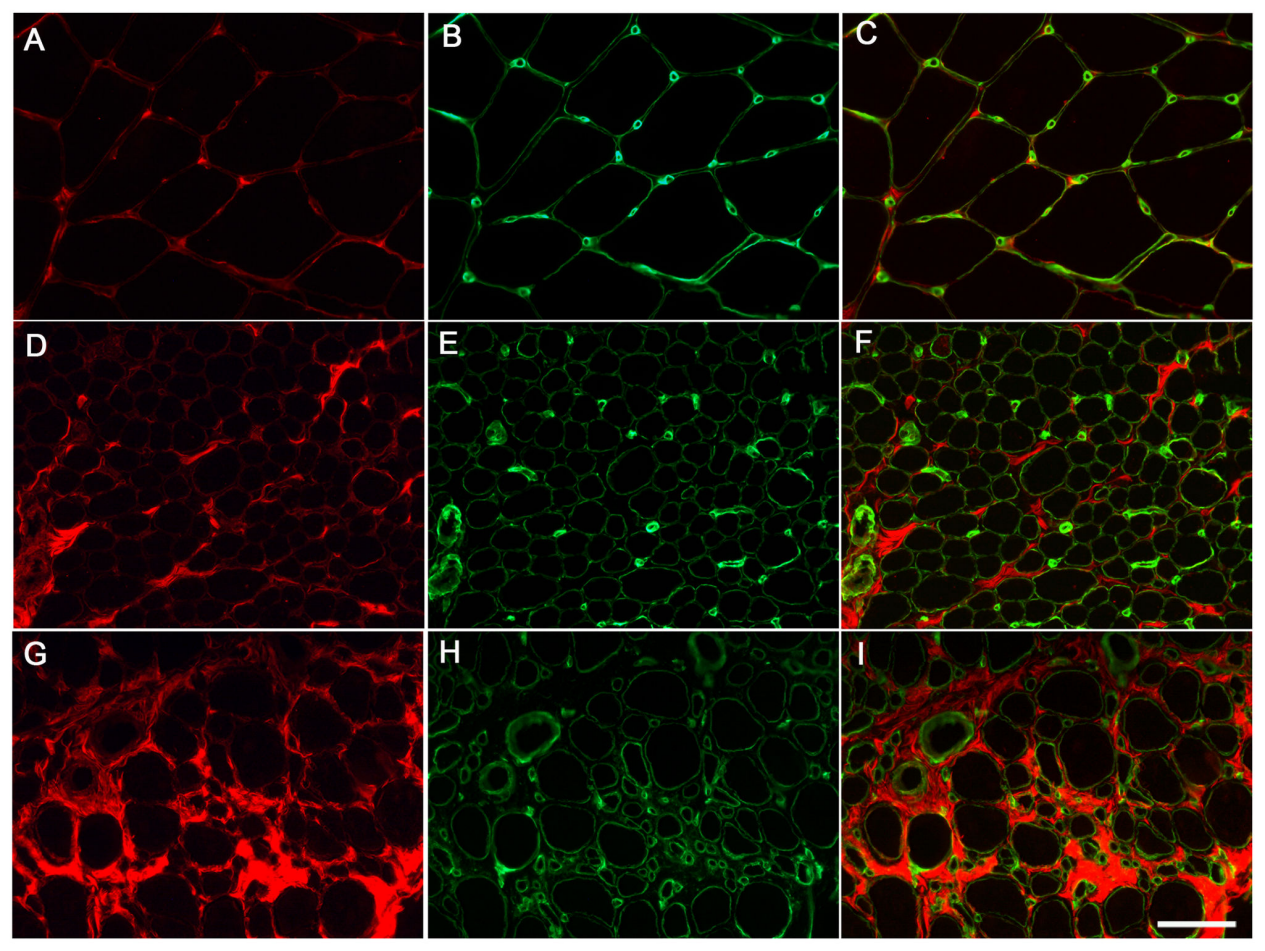
Biglycan immunolocalisation in UCMD muscle biopsies. Biglican (red) and perlecan (green) conventional immunofluorescence in muscle sections of controls (**A**-**C**), UCMD patient 9 (D-F) and patient 2 (G-I) showing a variable degree of biglican reduction. Scale bar: 50 µm.

**Figure 4:**
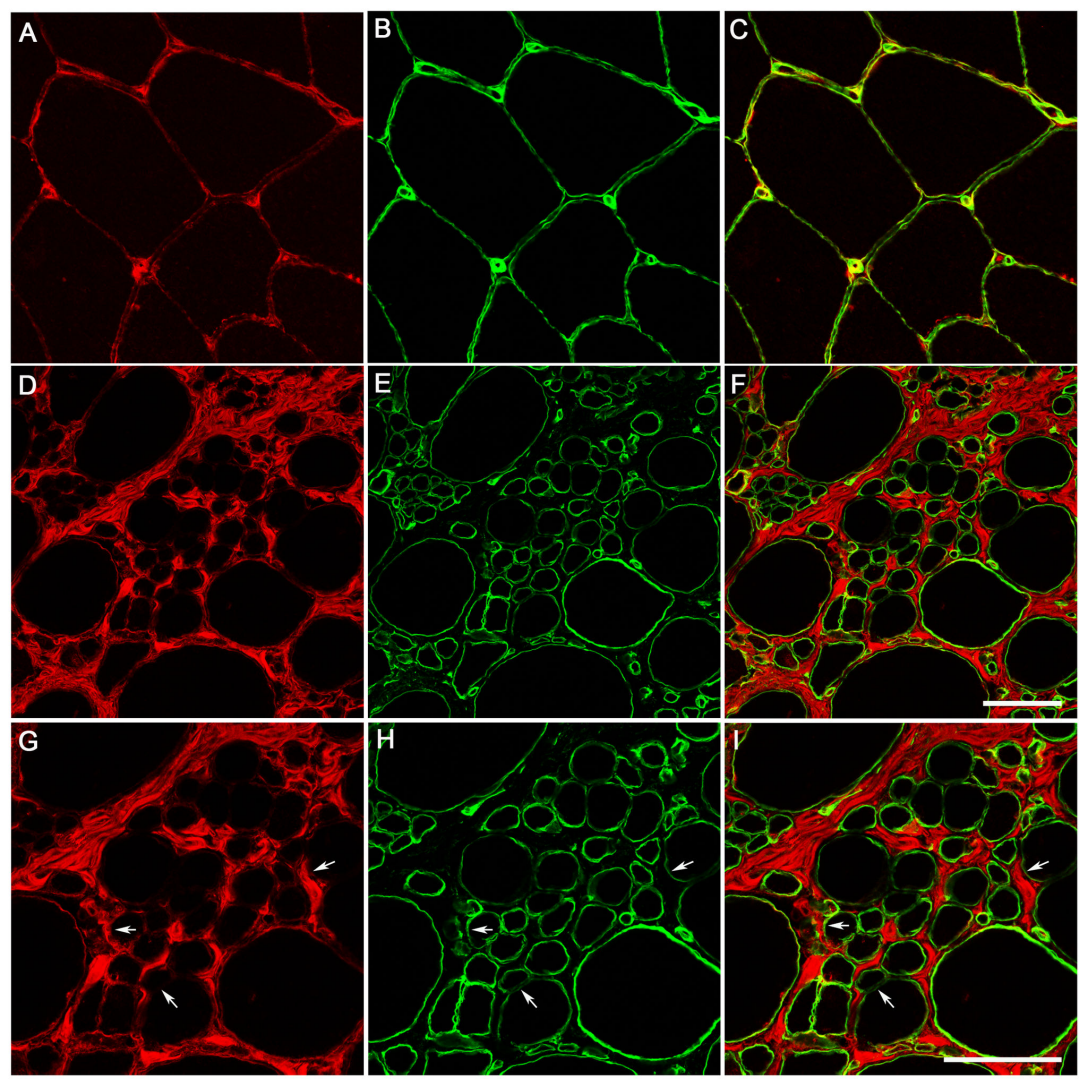
Secondary reduction of biglycan at the basal lamina of UCMD patients. The integrity of the basal lamina and the reduction of biglycan was further studied using confocal microscopy. An example for UCMD patient 6 at two different magnifications is shown. Arrows point to representative areas where biglycan (A, D, G) appears reduced relative to perlecan (B, E, H). Scale bar: 50 µm.

It has been described previously that in DMD and congenital muscular dystrophy 1A (MDC1A), biglycan expression at mRNA and protein level is up-regulated reflecting the extent of the fibrosis present in these dystrophies [35] [61]. However, changes in biglycan immunolocalisation have not been previously reported in UCMD. To investigate this we performed immunofluorescence against biglycan in muscle sections. In normal controls (n=6) biglycan was located predominantly in the perimysium and on the surface of the muscle fibres. In DMD muscle (n=3), biglycan was expressed similarly to controls except that the endomysial and perimysial compartments were more extensive, suggesting that presence of biglycan increases in relation to increased fibrosis in dystrophic muscle [58]. In UCMD muscles, biglycan was increased in the connective tissue, confirming microarray data. However, it was partially reduced at the muscle fibre basal lamina, relative to perlecan, in all 8 UCMD muscle samples analysed (7 genetically confirmed and one awaiting confirmation) compared to normal muscle. This specific reduction was confirmed using confocal microscopy. Representative images are shown in Figures 3 and 4. Given that collagen VI and biglycan are known to interact directly we hypothesise that the partial reduction in biglycan is secondary to collagen VI deficiency and may be contributing to the muscle dysfunction in UCMD . 

Previous studies have shown that biglycan over-expression regulates the sarcolemmal localization of α-sarcoglycan and γ-sarcoglycan [59], dystrobrevin [58], syntrophin, nNos [62] and utrophin [63] improving muscle integrity. Consequently, over-expression of biglycan is considered as a novel therapeutic target for DMD [63,64]. Similarly, over-expression of biglycan may restore localization of collagen VI at the basal lamina (where collagen VI reduction takes place in most UCMD patients) and thus strengthen binding of the muscle cell to the ECM improving muscle integrity and function in collagen VI deficiency.

Tenascin-X, an extracellular matrix glycoprotein which is produced by fibroblasts, was also up-regulated (+2.70). It is known that tenascin X interacts with types I, III and V collagen molecules and also binds to the fibril associated types XII and XIV collagens, therefore playing a crucial role in the organisation of ECM [65][66]. Moreover, tenascin X regulates the expression of type VI collagen at the transcriptional level promoting its synthesis [41]. Thus, up-regulation of tenascin X in UCMD muscles may be a compensatory mechanism to increase collagen VI production. In line with this we observed an increase of *COL6A1* and *COL6A6* mRNA expression (fold-change of 2.6 and 2.4, respectively) although *COL6A2* and *COL6A3* were not significantly changed in UCMD versus control muscle. 

Other components of the ECM such as Laminin alpha 4 (+3.38), Nidogen 2 (+2.73) and Fibulin 1 and 5 (+2.26) were also induced.

### Immune response

**Figure 5:**
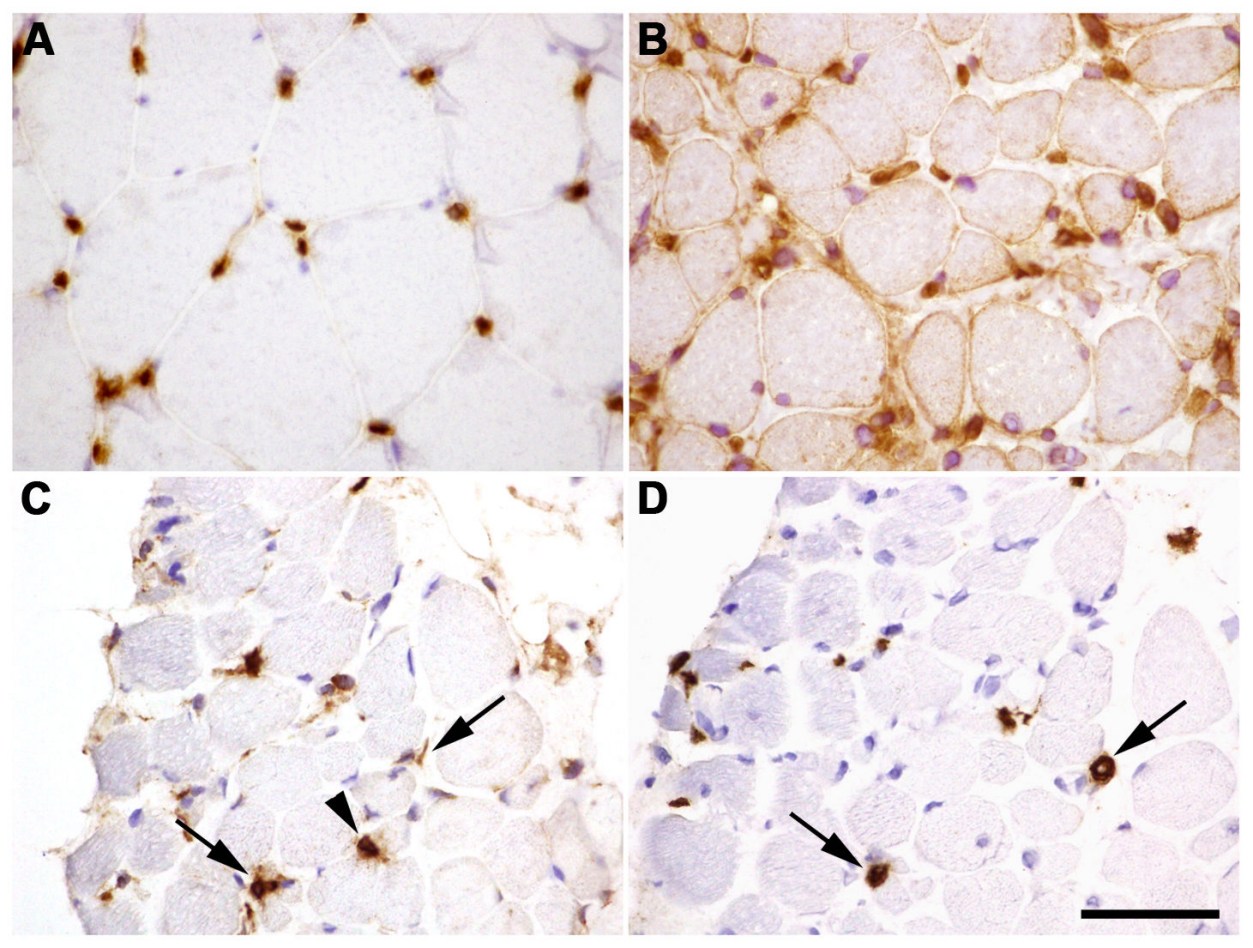
Characterization of inflammation found in UCMD muscle. HLA immunostaining in healthy muscle sections located in the endothelial cells of capillaries (**A**). Sarcolemmal and cytoplasmatic HLA staining on UCMD muscle sections including strong staining of mononucleated cells (**B**). Immunohistochemistry for CD68 demonstrate macrophage infiltration (**C**). Immunohistochemistry for CD206 identified M2-type macrophages (**D**). Arrows point to mononucleated cells CD68+ and CD206+ whereas arrowheads point to only CD68+ cells. Scale bar: 50 µm.

Our data indicate that collagen VI deficiency leads to activation of an immune response. We found increased expression of Class I and Class II Major Histocompatibility Complex (MHC) genes including beta-2-microglobulin (*B2M*, +2.3), *HLA-B* (+2.3) and *HLA-F* (+2.4). We investigated this further by immunohistochemistry for HLA-I antigen in UCMD muscle. We observed widespread HLA expression on the membrane of most muscle fibres and in the cytoplasm of some regenerating fibres, identified by perinatal myosin staining. In addition immunohistochemistry revealed inflammatory cell infiltrates in UCMD muscles. In contrast, in control muscle only capillaries and endothelial cells expressed HLA (Figure 5). Next, we examined inflammatory features in the skeletal muscle of UCMD patients. In control muscle, resident macrophages could be found in both the epimysium and the perimysium, but rarely found in the endomysium. However, in UCMD muscle we observed abundant endomysial infiltration and rarely perivascular inflammation by immune cells with morphological features resembling macrophages. 

To investigate the origin of inflammatory cells we performed immunohistochemistry against different cell markers including CD3, CD20 and CD68 that identify T cells, B cells and macrophages, respectively. We demonstrated that in UCMD the immune cells were macrophages, because they were positive for the macrophage marker CD68 and nor for the other cell markers. We corroborated this finding with acid phosphatase staining (data not shown). Various macrophage phenotypes could be present in muscle. ‘Classically activated’ macrophages (M1) produce both pro-inflammatory cytokines and nitric oxide, promoting muscle damage whereas ‘alternative activated’ macrophages (M2) are involved in muscle regeneration because they attenuate the inflammatory response and promote tissue repair. CD206 is a mannose receptor that is expressed by all M2 macrophages. In UCMD approximately 74% of CD68^+^ macrophages were also positive for the M2 type macrophage marker CD206 (Figure 4). Therefore, in UCMD muscle there is an important inflammatory component which consists mainly of M2 macrophages. In line with this *CCL-18*, which is an anti-inflammatory cytokine secreted by M2 macrophages, was also up-regulated (+3.19). 

It is not clear what triggers the inflammatory response in UCMD but over-expression of MHC-class I components suggest an autoimmune component which may be directed towards the abnormal collagen VI which is produced by these patients. Besides, the expression of components of the MHC-class II was also up-regulated in the microarrays, probably as a consequence of the presence of inflammatory cells in muscle. We determined that these inflammatory cells corresponded mainly to macrophages (CD68 positive) M2-type (CD260 positive), that promote tissue repair. Macrophage infiltration is thought to contribute to muscle wasting in DMD and their depletion or inhibition as been shown to significantly decrease dystrophic muscle pathology [67]. Glucocorticoids, more precisely prednisone and deflazacort, are the main drug treatment to DMD patients with established clinical efficacy in increasing muscle strength and slowing the progression of the illness [68]. It has been postulated that the benefit of corticosteroid treatment might be attributed to the anti-inflammatory as well as the immunosuppressive effect [69]. In view of our results, the potential beneficial effect of corticosteroid treatment in UCMD patients should be considered.

Our results indicate that classical as well as alternative complement pathways are activated and possibly contribute to muscle damage in UCMD. We identified the complement factors *C1R* (+2.30), *C1S* (+2.31), *C3* (+6.30), *C4B* (+2.48), *C7* (+2.49) and *CFD* (+4.58), as differentially expressed. In order to validate these results we measured the RNA levels of *C3*, a central component of the complement system, confirming its up-regulation in UCMD muscles. Complement system activation is not an exclusive feature of UCMD because it has been described in other muscular dystrophies [70] such as DMD [71], dysferlinopathy (LGMD2B) [72] and merosin deficiency (MDC1A) [73][74] where it is thought to contribute to membrane damage and macrophage recruitment amplifying in turn the immune response and muscle damage. In a mouse model of LGMD2B, other authors confirmed the active role of complement activation in the progression of muscular dystrophy and the attenuation of muscle pathology following the disruption of C3 providing a new therapeutic target for muscular dystrophies [75]. 

### Protein catabolism

70 genes were found significantly down-regulated in UCMD versus control muscle (Table 1). DAVID analysis identified 6 significant GO BP terms (with % FDR<20) to be associated to these down-regulated genes. All of them were related to protein degradation by the ubiquitin-proteasome system. These GO BP terms contained 7 unique genes: *USP31* (-2.60), *USP38* (-2.40), *ASB2* (-2.00), *NEDD4* (-2.20), *FBXO32* (-3.40) and *TRIM63* (-3.20). We had previously described down-regulation of two of these genes in UCMD muscle by means of qRT-PCR: *FBXO32* encoding the E3-ubiquitin ligase, atrogin-1 (MAFbx) and *TRIM63* encoding Muscle RING Finger-1 (MuRF1) [30]. 

In contrast, the autophagy-lysosomal system, another mechanism for protein degradation, was not regulated. Grumati et al. [12] have previously described defective autophagy in a collagen VI-knockout (Col6a1^-/-^) mice and human. Muscles from Col6a1^-/-^ mice showed reduced LC3-I to LC3-II conversion and had reduced levels of beclin-1 and Bnip-3 at protein level as well as in muscle biopsies derived from UCMD and BM patients. However, in our data we did not observe any changes in *BECN1* (Beclin-1) and *BNIP3* mRNA levels although a slight non-significant reduction of LC3-II (*MAP1LC3a*) was observed. Furthermore, non GO category or KEGG pathway related to autophagy was significant.

### Mitochondria and endoplasmic reticulum

Ultrastructural abnormalities in mitochondria and endoplasmic reticulum (enlarged and swollen organelles), mitochondrial dysfunction due to dysregulation of the permeability transition pore (PTP) and calcium homeostasis defects have been described in collagen VI knocked-out mice and UCMD muscles [9]. Previous works have described a higher incidence of apoptosis in biopsies from UCMD patients using TUNEL labelling [15,76]. In contrast, as we previously reported, in our group of UCMD patients we did not find evidence of muscle cell death by apoptosis (by active caspase-3 and TUNEL immunostaining) [30]. In accordance with our previous results, no significant functional categories related to apoptosis were identified in the present study.

However, GO (Table S5) and IPA analysis identified two canonical pathways related to calcium homeostasis as a significantly altered (Calcium-induced T Lymphocyte Apoptosis and Calcium Signaling with p-values 0.0018 and 0.0042, respectively) in UCMD muscle relative to normal muscle. We found down-regulation of SERCA1 (*ATP2A1*) (-1.9) suggesting decreased Ca^2+^ uptake by the sarcoplasmic reticulum. 

### Comparison with other muscular dystrophies

In order to identify UCMD specific gene expression patterns we also analysed other muscular pathologies including DMD, as a paradigm of muscular dystrophy, and other forms of congenital muscular dystrophies associated with either FKRP or laminin-α2 deficiency (CMD group, see materials and methods). We identified 718 (1195) genes (oligos) whose expression was changed significantly in UCMD versus DMD, 297 (643) of them were up-regulated and 421 (552) genes (oligos) were down-regulated (Table 1). 

Significant genes in this comparison together with fold changes are listed in Table S8. The down-regulated genes in UCMD versus DMD included some involved in regulation of transcription (*HMBOX1* and *SPEN*), cellular DNA damage response (*RPA4*), autophagy (*VPS18*), tumor suppression (*LRRC2*), *IGFN1*, *DDN* and a macrophage marker (*CD68*).

The most up-regulated genes were *HMGCS2*, one of the main enzymes for ketone-body synthesis, *ANGPTL4*, that mediates PPAR delta effect on fatty acid uptake, *RBP4*, an adipokine related to inflammation and insulin sensitivity and some molecules of major histocompatibility complex class II responsible of presenting peptides derived from extracellular proteins.

Bioinformatics analysis with DAVID comparing identified 122 significant GO BP terms, 115 of them for up-regulated genes and 7 for down-regulated ones (GO BP Categories were listed in Table S9). Similarly, 15 significant KEGG pathways were identified for up-regulated genes and 4 for down-regulated ones, listed in Table S10. 

These data indicated that markedly different pathways are involved in the pathophysiology of both types of muscular dystrophy.

Perhaps surprisingly only 29 (38) genes (oligos) were differentially under-expressed in UCMD compared to CMD (Table 1). Adequately, bioinformatics analysis with DAVID comparing UCMD with other CMD did not obtain any significant GO BP term nor KEGG pathway enriched theme (a full gene list is provided in Table S11). *CD86* was the most differentially expressed gene (-8.80). In other forms of CMD regeneration was more prominent than in UCMD as shown by the expression levels of embryonic myosin (*MYH3*, -5.35) and prominin 2 (also called *CD133*) a marker of muscle derived stem cells (*PROM2*, -4.55). Markers of oxidative stress (*GSTT1*) and autophagy (*VPS18*) were down-regulated in UCMD versus other forms of CMD.

Thus, these data suggest that in contrast to DMD, UCMD muscle shares several pathological mechanisms in common with other forms of CMD, at least at the transcriptional level. 

### Validation of array results with real-time quantitative PCR

The altered expression for 24 genes chosen from those identified in the microarray was analyzed by real-time PCR using the BioMark 48.48 Dynamic Arrays (Table 5). In total, we analysed 4 control muscle samples (Control 1, 2, 3 and 5, Table S1) and 7 UCMD muscle samples (UCMD 3, 4a, 4b, 5, 6, 7 and 8, Table S1) which include three additional samples not present in the microarrays. One of the most up-regulated genes in the microarray, *RBP4*, was one of these selected genes. Other genes were selected to represent the most regulated GO terms, i.e. regeneration (*TNNT2*), remodelling of the extracellular matrix (*COL14A1, COL19A1, COL21A1, DPT, LUM, MGP* and *TNXB*), inflammation and complement system (*CCL21, CXCL9* and *C3*), lipid metabolism and adipokines (*PPARG, HMGCS2, PLIN1, ADIPOQ* and *LEP*) and other representative down-regulated genes (*IGFN1* and *NEDD4*).

**Table 5:** Comparison of fold-changes obtained by microarray and by real-time PCR.

Gene	Microarray		Fluidigm	
	FC	q-value	FC	SE
IGFN1	-8.82	0	-18.83	± 0.48
NEDD4	-2.22	0.3464286	-2.43	± 0.08
TNXB	+2.70	0.03189453	+1.10	± 0.18
PPARG	+2.76	0.01098361	+2.36	± 0.17
COL21A1	+3.16	0.00699074	+3.36	± 0.38
LUM	+3.17	0.01145098	+1.73	± 0.45
MGP	+3.20	0.00757991	+1.87	± 0.48
CCL21	+3.31	0.00371429	+2.28	± 0.60
COL14A1	+3.34	0.02665944	+2.41	± 0.84
DPT	+3.37	0.0047449	+2.34	± 0.21
CXCL9	+3.72	0.00275362	+4.24	± 1.54
COL19A1	+4.13	0.00190909	+50.19	± 1.28
FST	+4.14	0.00155556	+5.64	± 0.38
CEBPA	+4.65	0.00111111	+6.19	± 0.19
MGST1	+5.06	0.00046900	+5.32	± 0.44
LEP	+5.10	0.00033300	+27.52	± 1.36
PTPRF	+5.69	0.00045500	+6.46	± 0.41
C3	+6.30	0.00045500	+5.28	± 0.37
TNNT2	+6.87	0.00045500	+15.07	± 0.64
HMGCS2	+7.50	0	+8.59	± 1.72
PLIN1	+9.45	0	+13.89	± 0.76
ADIPOQ	+9.61	0	+23.35	± 0.62
LGALS7	+10.89	0	+7.75	± 0.32
RBP4	+19.56	0	+18.63	± 0.64

FC: Fold-change; SE: standard error. Fold-changes were calculated as mean values of 2^- ∆∆CT^ or 1/2^- ∆∆CT^ relative to healthy controls. A fold-change above or below 1.5 was considered significant.

## Conclusions

This is the first report applying global gene expression analysis to identify biological functions and pathways involved in UCMD skeletal muscle pathophysiology. In addition to identifying the set of genes differentially expressed in UCMD versus normal muscle, we identified genes differentially expressed between UCMD and DMD and between UCMD and other forms of CMD. The few gene expression changes identified between the latter two groups indicate that the pathways implicated in distinct forms of CMD pathologies may be very similar. Therefore, the study of these genes and pathways could be helpful to better understand the mechanisms involved more generally in CMDs. 

Furthermore, we have identified a secondary reduction in biglycan in the basal lamina of collagen VI deficient muscle fibres. We hypothesise that biglycan over-expression could help restore the link between the basal lamina and the extracellular matrix and improve muscle pathology in UCMD. 

Additionally, we suggest that corticosteroid treatment and attenuation of the complement component 3 may help slow the progression of the disease although further research is required to elucidate the role of the immune system in the context UCMD.

## Supporting Information

Table S1 **List of patients.**
(DOCX)

Table S2 **List of Gene Expression Taqman Assays.**
(DOCX)

Table S3 **List of primary antibodies.**
(DOCX)

Table S4 **List of gene changes FDR<0.05 UCMD vs control.**
(XLSX)

Table S5 **List of significant GO BP categories in UCMD vs control muscle.**
(XLSX)

Table S6 **List of significant KEGG pathways in UCMD vs control muscle.**
(XLSX)

Table S7 **List of significant gene networks (IPA) in UCMD vs control.**
(XLSX)

Table S8 **List of gene changes FDR<0.05 in UCMD vs DMD.**
(XLSX)

Table S9 **List of significant GO BP categories in UCMD vs DMD.**
(XLSX)

Table S10 **List of significant KEGG pathways in UCMD vs DMD.**
(XLSX)

Table S11 **List of gene changes FDR<0.05 in UCMD vs CMD.**
(XLSX)

Figure S1 **Quantification of fibrosis.** Representative images of muscle sections of healthy control (**A**), UCMD patient (**B**) and DMD patient (**C**) immunolabelled against collagen type VI used to perform quantification of fibrosis with ImageJ software. Scale bar: 50 µm.
(TIF)
