# Correction: Gene Expression Profiling Identifies Molecular Pathways Associated with Collagen VI Deficiency and Provides Novel Therapeutic Targets

**DOI:** 10.1371/journal.pone.0128614

**Published:** 2015-05-14

**Authors:** Sonia Paco, Susana G. Kalko, Cristina Jou, María A. Rodríguez, Joan Corbera, Francesco Muntoni, Lucy Feng, Eloy Rivas, Ferran Torner, Francesca Gualandi, Anna M. Gomez-Foix, Anna Ferrer, Carlos Ortez, Andrés Nascimento, Jaume Colomer, Cecilia Jimenez-Mallebrera

There are errors in the Funding section. The correct funding information is as follows: This work is funded by the “Plan Nacional de I+D+I and Instituto de Salud Carlos III- Subdirección General de Evaluación y Fomento de la Investigación Sanitaria", project PI10/00177, and the European Regional Development Fund (FEDER). The funders had no role in study design, data collection and analysis, decision to publish, or preparation of the manuscript.

## References

[1] Paco S, Kalko SG, Jou C, Rodríguez MA, Corbera J, Muntoni F, et al (2013) Gene Expression Profiling Identifies Molecular Pathways Associated with Collagen VI Deficiency and Provides Novel Therapeutic Targets. PLoS ONE 8(10): e77430 doi: 10.1371/journal.pone.0077430 24223098 10.1371/journal.pone.0077430 PMC3819505

