# Supplementary material for: Gene Expression Profiling Identifies Molecular Pathways Associated with Collagen VI Deficiency and Provides Novel Therapeutic Targets
Source: PLoS One. 2013 Oct 11;8(10):e77430. doi: 10.1371/journal.pone.0077430 (PMC3819505; doi:10.1371/journal.pone.0077430)
Supplement: Table S1 — List of patients. (DOCX) [file pone.0077430.s001.docx]

**Table S1.** List of patients.

|  | **Gender** | **Age** | **Diagnosis** | **Mutation** | **Microarray** | **Validation** |
| --- | --- | --- | --- | --- | --- | --- |
| **C1** | M | 6 y | - | - | √ | √ |
| **C2** | M | 13 y | - | - | √ | √ |
| **C3** | M | 13 y | - | - | √ | √ |
| **C4** | M | 1 y | - | - | √ | - |
| **C5** | M | 16 y | - | - | - | √ |
| **P1** | M | 3 y | UCMD | COL6A1 exon 10: c.877G>A (p.Gly293Arg) Heterozygous | √ | - |
| **P2** | F | 3 y | UCMD | COL6A1 exon 10: c.877G>A (p.Gly293Arg) Heterozygous | √ | - |
| **P3** | M | 4 y | UCMD | COL6A1 exon 10: c.877G>A (p.Gly293Arg) Heterozygous | √ | √ |
| **P4a** | M | 5 y | UCMD | COL6A1 aberrant transcript joining exon 18 to exon 25 coexisting with a normal transcript in cDNA from fibroblasts | √ | √ |
| **P4b** | M | 7 y | UCMD | COL6A1 aberrant transcript joining exon 18 to exon 25 coexisting with a normal transcript in cDNA from fibroblasts | √ | √ |
| **P5** | F | 9 y | UCMD | Underway | √ | √ |
| **P6** | M | 3 y | UCMD | COL6A3 intron 18: c.6283+3 A>G (pGly2095_Lys2103del) Heterozygous | - | √ |
| **P7** | F | 2 y | UCMD | COL6A2 intron 9: c.955-2 A>G Heterozygous | - | √ |
| **P8** | F | 8 y | UCMD | COL6A1 intron 5: c.717+4 A>G (p.Ile239fsX30) Homozygous | - | √ |
| **P9** | F | 1,5 y | UCMD | COL6A2 exon 7: (Gly298Arg) Heterozygous | - | - |
| **DMD1** | M | 4 y | DMD | Deletion exons 48-52 DMD | √ | √ |
| **DMD2** | M | 2 y | DMD | Deletion exon 50 DMD | √ | - |
| **DMD3** | M | 6 y | DMD | * | √ | √ |
| **CMD1** | F | 1 y | MDC1A | ** | √ | √ |
| **CMD2** | M | 1 y | MDC1A | ** | √ | √ |
| **CMD3** | F | 14 y | MDC1C | FKRP 265C>T (Pro89Ser) and 1443T>G (Ile478Ser) Heterozygous | √ | - |

Abbreviations: M: male; F: female; Y: year; UCMD: Ullrich Congenital Muscular Dystrophy. DMD: Duchenne Muscular Dystrophy; MDC1A: Muscular Dystrophy Congenital Type 1 A; MDC1C: Muscular Dystrophy Congenital Type 1 C.*: Diagnosis of DMD in this patient was based on complete dystrophin deficiency and clinical findings. ** MDC1A in these patients is based on complete laminin-α2 absence on muscle biopsy and clinical findings
