# Supplementary material for: Gene Expression Profiling Identifies Molecular Pathways Associated with Collagen VI Deficiency and Provides Novel Therapeutic Targets
Source: PLoS One. 2013 Oct 11;8(10):e77430. doi: 10.1371/journal.pone.0077430 (PMC3819505; doi:10.1371/journal.pone.0077430)
Supplement: Table S2 — List of Gene Expression Taqman Assays. (DOCX) [file pone.0077430.s002.docx]

**Table S2.** List of Gene Expression Taqman Assays.

| **Gene name** | **Gene symbol** | **Taqman Gene Expression Assays** |
| --- | --- | --- |
| TATA box binding protein | TBP | Hs99999910_m1 |
| Hypoxanthine phosphoribosyltransferase 1 | HPRT1 | Hs02800695_m1 |
| Immunoglobulin-like and fibronectin type III domain containing 1 | IGFN1 | Hs00297580_m1 |
| Lectin, galactoside binding, soluble, 7 | LGALS7 | Hs00170104_m1 |
| Matrix Gla protein | MGP | Hs00179899_m1 |
| Retinol binding protein 4 | RBP4 | Hs00924047_m1 |
| 3-hydroxy-3-methylglutaryl-CoA synthase 2 (mitochondrial) | HMGCS2 | Hs00985427_m1 |
| Collagen, type XIV, alpha1 | COL14A1 | Hs00385388_m1 |
| Complement component 3 | C3 | Hs00163811_m1 |
| Perilipin 1 | PLIN1 | Hs00160173_m1 |
| Lumican | LUM | Hs00929860_m1 |
| Collagen, type XIX, alpha1 | COL19A1 | Hs00156940_m1 |
| Collagen, type XXI, alpha1 | COL21A1 | Hs00229402_m1 |
| Tenascin XB | TNXB | Hs00372889_g1 |
| Dermatopontin | DPT | Hs00355056_m1 |
| Protein tyrosine phosphatase, receptor type, F | PTPRF | Hs00892965_m1 |
| Peroxisome proliferator-activated receptor gamma | PPARG | Hs01115513_m1 |
| Chemokine (C-C motif) ligand 21 | CCL21 | Hs00989654_g1 |
| Chemokine (C-X-C motif) ligand 9 | CXCL9 | Hs00171065_m1 |
| Neural precursor cell expressed, developmentally down-regulated 4, E3 ubiquitin protein ligase | NEDD4 | Hs00406454_m1 |
| Follistatin | FST | Hs00246256_m1 |
| Troponin T type 2 (cardiac) | TNNT2 | Hs00165960_m1 |
| Adiponectin, C1Q and collagen domain containing | ADIPOQ | Hs00605917_m1 |
| Leptin | LEP | Hs00174877_m1 |
| CCAAT/enhancer binding protein (C/EBP), alpha | CEBPA | Hs00269972_s1 |
| Microsomal glutathione S-transferase 1 | MGST1 | Hs00220393_m1 |
