# Supplementary material for: Gene Expression Profiling Identifies Molecular Pathways Associated with Collagen VI Deficiency and Provides Novel Therapeutic Targets
Source: PLoS One. 2013 Oct 11;8(10):e77430. doi: 10.1371/journal.pone.0077430 (PMC3819505; doi:10.1371/journal.pone.0077430)
Supplement: Table S3 — List of primary antibodies. (DOCX) [file pone.0077430.s003.docx]

**Table S3.** List of primary antibodies.

| **Antigen** | **Source** | **Cat. Nº** | **Working dilution** |
| --- | --- | --- | --- |
| Perlecan | Millipore | AB1948 | 1:5000 |
| Biglycan | Abcam | Ab54855 | 1:100 |
| HLA | Dako | M0736 | 1:1200 |
| CD68 | DBS | Mob167 | 1:2000 |
| CD206 | BD Pharmingen | 555953 | 1:150 |
| Collagen VI | Millipore | MAB1944 | 1:25 |
