# Supplementary figures and images for: Gene Expression Profiling Identifies Molecular Pathways Associated with Collagen VI Deficiency and Provides Novel Therapeutic Targets
Source: PLoS One. 2013 Oct 11;8(10):e77430. doi: 10.1371/journal.pone.0077430 (PMC3819505; doi:10.1371/journal.pone.0077430)

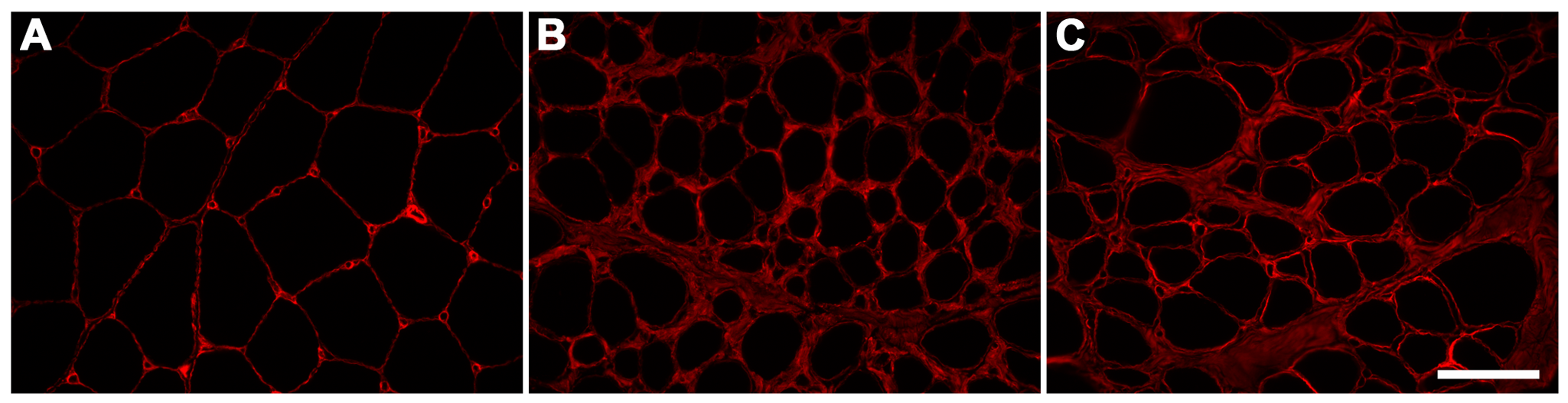

Supplement: Figure S1 — Quantification of fibrosis. Representative images of muscle sections of healthy control (A), UCMD patient (B) and DMD patient (C) immunolabelled against collagen type VI used to perform quantification of fibrosis with ImageJ software. Scale bar: 50 µm. (TIF) [file pone.0077430.s012.tif]
